# Effects of irrigation type and fertilizer application rate on growth, yield, and water and fertilizer use efficiency of silage corn in the North China Plain

**DOI:** 10.7717/peerj.18315

**Published:** 2024-11-27

**Authors:** Yuchun Liu, Ning Wang, Changsong Jiang, Yao Wang

**Affiliations:** 1 College of Urban and Rural Construction, Hebei Agricultural University AND Key Laboratory for North China Water-Saving Agriculture of Agriculture and Rural Affairs Ministry, Baoding, Hebei, China; 2 College of Urban and Rural Construction, Hebei Agricultural University, Baoding, Hebei, China

**Keywords:** Irrigation type, Fertilizer rate, Silage corn, Water and fertilizer use efficiency, North China Plain

## Abstract

**Background:**

There is an urgent need to save water and decrease fertilizer application rates in agricultural areas of the North China Plain (NCP) and similar regions.

**Methods:**

Field experiments were conducted in 2017 and 2018 in the NCP to investigate the effects of border and furrow irrigation under different fertilizer application rates on the growth, yield, and water and fertilizer use efficiencies of silage corn. The experiment applied two irrigation methods, i.e., border and furrow irrigation, each with four compound fertilizer application rates: 750, 600, 450, and 300 kg/ha.

**Results:**

While the two experiment years were normal hydrological years, variation in precipitation resulted in no irrigation being applied in 2017 and 70 mm of irrigation being applied after sowing in 2018. Plants appeared to grow slightly taller and thicker with larger leaf areas, but with a 9.7% lower fresh weight yield in 2017 relative to 2018. The actual evapotranspiration (ET_a_) in 2017 was 37.22 mm more than that in 2018, and the higher fresh weight yield and lower ET_a_ caused higher water use efficiency (WUE) in 2018, which was 32% higher than that in 2017. Furrow irrigation promoted growth compared with border irrigation under the same irrigation amount, but without significant effects on fresh weight yield, ET_a_, or WUE of silage corn. The fertilizer application amount had a significant effect on fresh weight yield and the partial fertilizer productivity of N, P and K of silage corn, but did not affect ET_a_ or WUE of silage corn. Additionally, the fertilizer rate of 600 kg/ha induced slightly higher growth indexes and fresh weight yields compared with the fertilizer rates of 750, 450, and 300 kg/ha.

**Discussion:**

In the NCP, lower irrigation amounts at the crop development period of silage corn appeared to promote higher yield, WUE, and fertilizer use efficiency, under the condition that there was sufficient water to ensure the emergence of seedlings. The current fertilizer application model, compound fertilizer applied with a seeder at planting, does not match the fertilizer needs of silage corn, and more efficient water and fertilizer application techniques should thus be adopted.

## Introduction

The North China Plain (NCP), located in the east of China (112°30′E to 119°30′E, 34°46′N to 40°25′N), covers 0.31 million km^2^. The NCP, as the political and cultural center of China, has been subjected to long-term intensive cultivation, remains one of the most important agricultural areas, and serves as the national agricultural base and major grain production region of China (Chen et al., 2020; Du et al., 2021). The winter wheat and summer maize double-cropping system is cultivated on 60% of the arable land in the NCP and contributes more than one half and one third of the total wheat and maize production, respectively, in China (Dan et al., 2020). Groundwater plays an important role in supporting agriculture, industry, and human well-being, and the overall available fresh groundwater reaches 1.92 × 10^10^ m^3^ y^−1^ in the NCP. However, the water resources in the NCP are merely 456 m^3^ y^−1^ per capita, which is far less than the internationally recognized standard for water resource shortages, 1,000 m^3^ y^−1^ per capita, and is below one seventh of the national average and one twenty-fourth of the world average (Chen et al., 2020).

The irrigation sector is the largest consumer of water, and numerous studies have been conducted to address the challenge of water scarcity in the face of climate change (Mubarik et al., 2020; Merrium et al., 2022). Because of water scarcity, the irrigation and water conservancy facilities of NCP farmlands are well equipped. Low-pressure pipeline water distribution is widely used, and surface irrigation, especially border irrigation, is widely adopted in the field. Suitable water-saving irrigation technologies, like furrow and drip irrigation, are recommended for winter wheat and corn production in the NCP (Bai, Kang & Wan, 2020b; Bai, Kang & Wan, 2020a; Wang et al., 2020; Yang et al., 2019; Sui et al., 2015). The limited availability of irrigation water in the NCP has motivated the adoption of suitable water-saving irrigation technologies, and furrow irrigation and border irrigation are promising alternative irrigation technologies that merit comparative study in the NCP. Under limited irrigation conditions, maize or onion can be irrigated with alternate furrow irrigation methods to maximize their water use efficiency (Akele, 2019; Jemal & Berhanu, 2020). Compared to conventional furrow irrigation, alternative furrow irrigation can increase nitrate-N contents in the upper soil layer (0–60 cm) and reduce nitrate-N movement to deeper soil, suggesting it may be an appropriate irrigation method for improving nitrogen use efficiency (NUE) and decreasing water consumption in winter wheat and dry season maize without reducing yields (Dianyong et al., 2021; Sarker et al., 2020). Additionally, alternate furrow irrigation uses up to 25% less water, provides comparable yields, and enhances water use efficiency (WUE) and economic benefits in irrigating potato crops (Kassaye et al., 2020). The results of the decomposition analysis of income differences between furrow and border irrigation in the cultivation of wheat showed that the adopters of border strip irrigation produced 24.87% higher incomes relative to the use of furrow irrigation (Rudrapur, Patil & Yeledhalli, 2014).

Another crop production issue in the NCP is its high fertilizer use. Attributed to overuse of chemical fertilizer, ignorance of the contribution of nutrients from the environment and soil, failure to achieve full crop yield potential, and the inability to inhibit nutrient leaching effectively, the apparent nitrogen recovery efficiency of wheat and maize were estimated to be 28.2% and 26.1%, respectively, in China, substantially much lower than the worldwide averages (Zhang et al., 2008). Long-term nitrogen fertilizer application can affect soil nitrogen processes by increasing soil nitrogen content and reducing the contribution of nitrogen from fertilizer (Joris et al., 2020). Deep soil cores from experimental plots in the NCP where N-fertilizer has been applied for nearly 20 years under wheat–maize double cropping indicate that the large N stock and generally weak denitrification potential in the deep unsaturated zone pose a major threat to future groundwater quality (Wang et al., 2019). Thus, water conservation and reduced fertilizer use are policies being pursued by the Chinese government to increase WUE and fertilizer use efficiency and protect the ecological environment. Reducing fertilizer applications by 10% to 20% may not reduce yield but indeed increase nitrogen use efficiency of crops (Tu, Zhang & Liu, 2021; Kenawy, Hosny & Saad-Allah, 2020). Many replaced fertilizers have been adopted to reduce mineral fertilizer use, including urea fertilizer (Kenawy, Hosny & Saad-Allah, 2020), organic fertilizer (Xie et al., 2021), stabilized nitrogen fertilizer (Mateo-Marín, Quílez & Isla, 2020), among others.

In the present study, experiments were conducted in 2017 and 2018 on silage corn under surface irrigation and different fertilizer application rates in the NCP. This study provides an empirical foundation and technical support for water and fertilizer management in silage corn production in the NCP and agricultural regions facing similar constraints. The experimental field in this study was planted by an agricultural planting company with conventional local planting patterns, with four different fertilizer application rates, to elucidate the effects of fertilizer reduction.

## Materials and Methods

### Study area

Field experiments were conducted in Nanlonggui village, Shijiazhuang, Hebei, China in 2017 and 2018. The experiment corn was sown at the end of June or early July and harvested at the end of September. The last crop was winter wheat, sown in early October and harvested in early June of the following year. The study area, which has a semi-humid continental monsoon climate, is flat, 122 m above sea level, and located at 37°56′54.1″N, 114°25′49.6″E. The yearly average sunshine duration, air temperature, precipitation total, and evaporation of the experiment region are 2,554 h, 12.2 °C, 536 mm, and 1,610.1 mm respectively. The soil of the study area is loam throughout the top 100 cm layer, and the physical and hydraulic properties of the experiment soils are summarized in Table 1. Only the initial nutrient contents at a depth of 0–20 cm were taken and summarized in Table 2. The initial nutrient contents at the other depth were not taken.

**Table 1:** Physical and hydraulic properties of experimental soils.

Soil depth (cm)	Bulk density (g cm^−3^ )	Soil particle grading (USA)			Texture	Saturated water content (cm^3^ cm^−3^)	Field capacity (cm^3^ cm^−3^)
		Sand (%)	Silt (%)	Clay (%)			
0–20	1.25	47.42	50.38	2.20	Loam	0.302	0.232
20–40	1.33	38.47	58.98	2.55	Silty loam	0.390	0.255
40–60	1.33	43.09	56.33	2.98	Clay loam	0.365	0.258
60–80	1.29	40.69	54.23	2.68	Silty clay loam	0.418	0.276
80–100	1.43	40.6	56.60	2.80	Clay loam	0.432	0.318

**Table 2:** Initial nutrient content of experimental soils at a depth of 0–20 cm.

Year	Organic matter	Alkaline hydrolysis nitrogen/mg/kg	Nitrate nitrogen/mg/kg	Readily phosphorus/mg/kg	Readily available potassium/mg/kg
2017	3%	126	26.8	13.21	131
2018	2%	137	39.2	11.89	158

### Experimental design

In 2017, irrigation was not applied because sufficient precipitation occurred during the growth period of silage corn, and the fertilizer application rate was 600 kg ha^−1^ for the experiment field. In 2018, irrigation type and fertilizer rate were experimental variables. Two irrigation types were adopted, border and furrow irrigation. Compound fertilizer application rates of 750, 600, 450, and 300 kg ha^−1^ were used in the experiments, based on growers’ standard rates and experimental rates for decreasing fertilizer application rates in China. The compound fertilizer (28% N, 6% P_2_O_5_, and 6% K_2_O) and silage corn seeds were applied at the same time with a seeder, and no other fertilizer was applied for the duration of the experiment. There were eight experimental treatments with three replicates each, for a total of 24 experiment plots. The experimental plots were laid out in a randomized complete block design.

The soil of the experiment field was prepared before sowing by laser leveling (1JP350) and the slope of the soil preparation was 0.5‰. The corn variety used in the experiments was Keyu 188, which is a variety commonly grown in the study region. The planting density was 78 thousand plants ha^−1^, which is a typical grower standard in the study area. The rows were spaced 60 cm apart, and the distance between the plants cultivated along the lines was 30 cm. The experiment plots were separated by 1.5 m to ensure that the treatments were independent of each other and regularly weeded and treated for pests and diseases during the growth period.

In 2018, the border width was 4.8 m, as determined by the standard widths of common agricultural machinery, including seeders, pesticide applicators, and harvesters. The border length was 90 m, with nine rows of silage corn planted within each border. The furrows were constructed with a ditcher and artificially shaped. In each experimental plot, there were eight furrows, 90 m in length and separated by 60 cm. The widths of the top and bottom of each furrow were 40 and 20 cm, respectively, and each furrow was 20 cm deep. Both the experimental borders and furrows had closed ends. The flow rate of the well was 35 m^3^/h, with one border or plot irrigated under border irrigation, and the unit flow per meter width was 2.03 L/s. The stream size per furrow was 1.22 L/s under furrow irrigation, and eight furrows were irrigated together.

**Figure 1:**
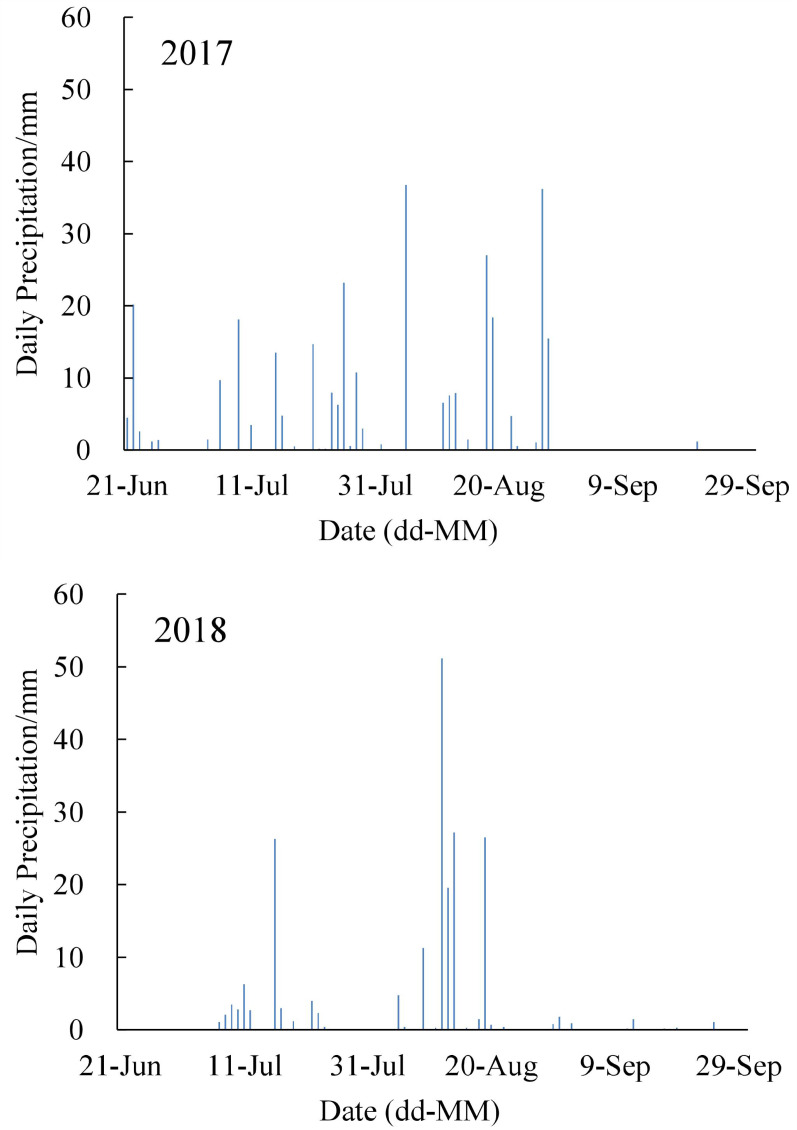
The precipitation during the growth period of silage corn in 2017 and 2018.

The average annual precipitation in the study area was 401.1–595.9 mm. The annual precipitation was 484.2 mm in 2017 and 509.2 mm in 2018, and the two years were both relatively normal precipitation years. In 2017, silage corn was planted on July 2 and harvested on September 30, while planting and harvesting occurred on June 27 and September 30, respectively, in 2018; thus, the experiment duration was 91 d in 2017 and 95 d in 2018. The precipitation during the growth period of silage corn was 284.6 mm in 2017 and 206.9 mm in 2018 (Fig. 1); thus, there was 77.7 mm more precipitation during the growth period of silage corn in 2017 relative to 2018. There was 41.1 mm of precipitation from June 21 to July 10 in 2017, which provided sufficient water to meet the requirements for the seeding stage of silage corn, and thus, no irrigation was applied. Owing to the low soil water content at sowing and the lack of precipitation from June 21 to July 10 in 2018, the field was irrigated with 70 mm of water immediately after sowing on June 28 to maximize the emergence rate, and no other irrigation was required for the duration of the experiment owing to adequate precipitation.

### Measurement methods and calculations

To measure the initial soil water content before sowing, soil samples were collected using a soil drilling method at three randomly selected points at depths of 0–20, 20–40, 40–60, 60–80, and 80–100 cm. During the growth period, the rows of silage corn with proper density and uniform growth were selected for sample collection to measure soil water content, and the soil samples were collected from between corn plants within the rows. Soil samples were collected every 8 days, and additional samples were taken after irrigation and precipitation. Four sections along the length of the border or furrow, at distances of 9, 33, 57, and 81 m from the water inlet, were selected for sampling, and the average soil water content of these four sections was used to calculate water consumption of silage corn for the different growth periods. The soil water content was measured from these soil samples using the oven method.

During the silage corn growth period, plant samples were collected every 7–8 days to monitor plant growth traits, which included plant height, stem diameter, leaf area index (LAI), and chlorophyll content. The fresh yield of silage corn was recorded at harvest. During sampling conducted for observation of growth and yield, 3–5 plants with good and consistent growth were randomly selected from each experimental plot. Plant height was measured with a box ruler from the ground to the highest part of the plant. The stem diameter was measured with 111N-101 electronic Vernier calipers at 3–5 cm above the ground. The chlorophyll content was measured using the TYS-A chlorophyll analyzer. The leaf area of silage corn was measured according to the Montgomery equation (Chang, 1968) before the seven-leaf stage. The longest and widest parts of the blade were measured with the box ruler to an accuracy of one mm, and the single leaf area was estimated as the maximum length of the blade multiplied by the maximum width of the blade and then multiplied by a leaf area coefficient of 0.75. The leaf area of a plant was estimated as the sum of every single leaf area. The Pearce method (Pearce, Mock & Bailey, 1975) was used to determine the leaf area of a single plant after the jointing stage. The leaf area of the 8th leaf from the top to the bottom of the plant was determined by using the above Montgomery method, and the leaf area of a plant was the leaf area of the 8th leaf multiplied by 9.39. LAI estimates were calculated using Eq. (1).

(1) \begin{eqnarray*}\mathrm{LAI}= \left[ \rho \times \alpha \sum _{i=\mathrm{l}}^{n}({L}_{i}\cdot {W}_{i}) \right] \left/ \right. A.\end{eqnarray*}

Here, *ρ* is the plant density of the experimental plot (plant number/every experimental plot), *α* is the correction coefficient of silage corn leaf area (*α* = 0.75), *L*_*i*_ and *W*_*i*_ are the maximum length and width of the leaf (in cm), *n* is the number of leaves of the measured plant, and *A* is the area of the experiment plot (in cm^2^). The fresh weight yield of silage corn was measured with an electronic balance with a precision of 0.01 g at harvest.

The reference evapotranspiration ET_0_ was calculated from meteorological data by the Penman–Monteith method as follows (Allen et al., 2006):

(2) \begin{eqnarray*}E{T}_{0}= \frac{0.408\Delta \left( {R}_{n}-G \right) + \frac{900}{T+273} \gamma {U}_{2} \left( {e}_{s}-{e}_{a} \right) }{\Delta +\gamma \left( 1+0.34{u}_{2} \right) } .\end{eqnarray*}

Here, *ET*_0_ is the reference evapotranspiration (in mm day^−1^), *R*_*n*_ is the net radiation at the crop surface (MJ m^−2^ day^−1^), *G* is the soil heat flux density (in MJ m^−2^ day^−1^), *T* is the mean daily air temperature at a 2-m height (in ^∘^C), *u*_2_ is the wind speed at a 2-m height (m s^−1^), *e*_s_ is the saturation vapor pressure (in kPa), *e*_a_ is the actual vapor pressure (in kPa), (*e*_s_ - *e*_a_) is the saturation vapor pressure deficit (in kPa), △“-”- is the slope vapor pressure curve (in kPa^∘^C^−1^), and *γ* is the psychrometric constant (in kPa C^−1^).

The growth period of silage corn was separated into three periods, *i.e.,* crop development, mid-season, and late-season periods, and the actual evapotranspiration ET_a_ for each of these three growth periods was calculated by the field water balance in the root zone, as follows:

(3) \begin{eqnarray*}E{T}_{a}={P}_{0}+M- \left( {W}_{t}-{W}_{0} \right) -R-D+K.\end{eqnarray*}

Here, ET_a_ is the actual evapotranspiration of silage corn (in mm day^−1^); *P*_0_ is the effective rainfall (in mm), where *P*_0_ = *σP* and *σ* = 0 when *P* < 5 mm, *σ* = 1 when 5 ≤*P* ≤ 50 mm, and 0.7 ≤*σ* ≤ 0.8 when *P* >  50 mm; *M* is the irrigation amount (in mm); *W*_0_ and *W*_*t*_ are the soil water storage in the root zone at the beginning and end of a growth period (in mm), as calculated based on the measured soil water content during the growth period; *R* is the surface runoff (in mm), assumed to be negligible owing to the flat topography of the study area; *D* is deep percolation below the 1-m deep root layer (in mm), assumed to be 10% of the sum of effective rainfall (*P*_0_) and irrigation amount (*M*) according to related research results on deep percolation in fields with greater groundwater depth (Du & Shao, 2013; Liu et al., 2015); and *K* is the groundwater recharge (in mm), assumed to be zero owing to the deeply buried underground water level of the study area. The crop coefficient *K*_*c*_ is the ratio of ET_a_ and ET_0_, and the crop coefficient of silage corn at the crop development, mid-season, and late-season crop periods, as well as the whole growth period, were calculated.

WUE was calculated based on fresh weight yield (*Y*) and ET_a_. Partial fertilizer productivity of N (PFPN), P (PFPP), and K (PFPK) of silage corn were calculated based on the fresh weight yield (*Y*) and fertilizer application amount of N, P, and K.

(4) \begin{eqnarray*}WUE& =Y/E{T}_{a}\end{eqnarray*}

(5) \begin{eqnarray*}PFPN& =Y/N\end{eqnarray*}

(6) \begin{eqnarray*}PFPP& =Y/P\end{eqnarray*}

(7) \begin{eqnarray*}PFPK& =Y/K.\end{eqnarray*}

### Statistical analysis

In 2017 a uniform fertilizer application rate of 600 kg ha^−1^ was used in the experiment, and thus, a fertilizer rate of 600 kg ha^−1^ treatment under border irrigation was used when the results of the two annual trials were compared. Correlations between fresh weight yield, plant height, stem diameter, LAI, and leaf SPAD value were examined to explore the factors affecting silage corn. ANOVA was used to analyze the effects of irrigation type and fertilizer amount on the growth, water consumption amount, WUE, and fertilizer use efficiency of silage corn.

## Results

### Effects on growth and yield

**Figure 2:**
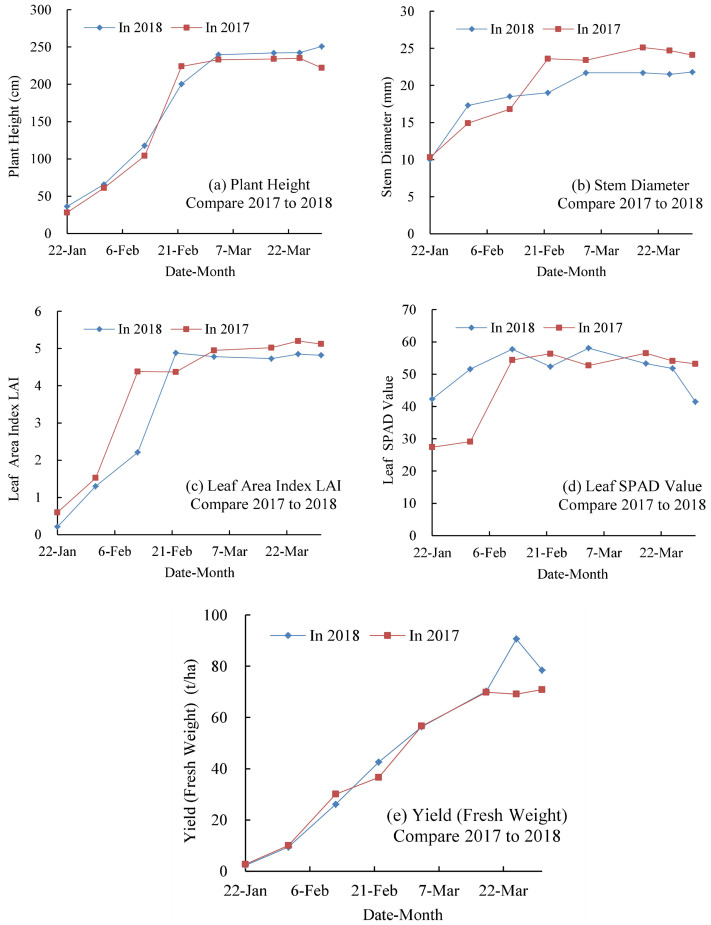
Plant height, stem diameter, leaf area index (LAI), SPAD value, and fresh weight yield of silage corn throughout the growing period in 2017 and 2018.

The comparison of plant height, stem diameter, LAI, leaf SPAD value, and fresh weight yield in the experiment years 2017 and 2018 are shown in Fig. 2. The height and LAI increased linearly from sowing to about 50 days after sowing and then slightly increased afterwards. The stem diameter and fresh weight gradually increased from sowing to about 80 days after sowing and then slowly decreased until harvest. The stem diameter in 2017 was 10.6% greater than that in 2018, and the LAI in 2017 was 6.2% greater than that in 2018 owing to the higher precipitation total occurring during the growing season in 2017. However, the fresh weight yield in 2017 was 9.7% lower than that in 2018.

**Figure 3:**
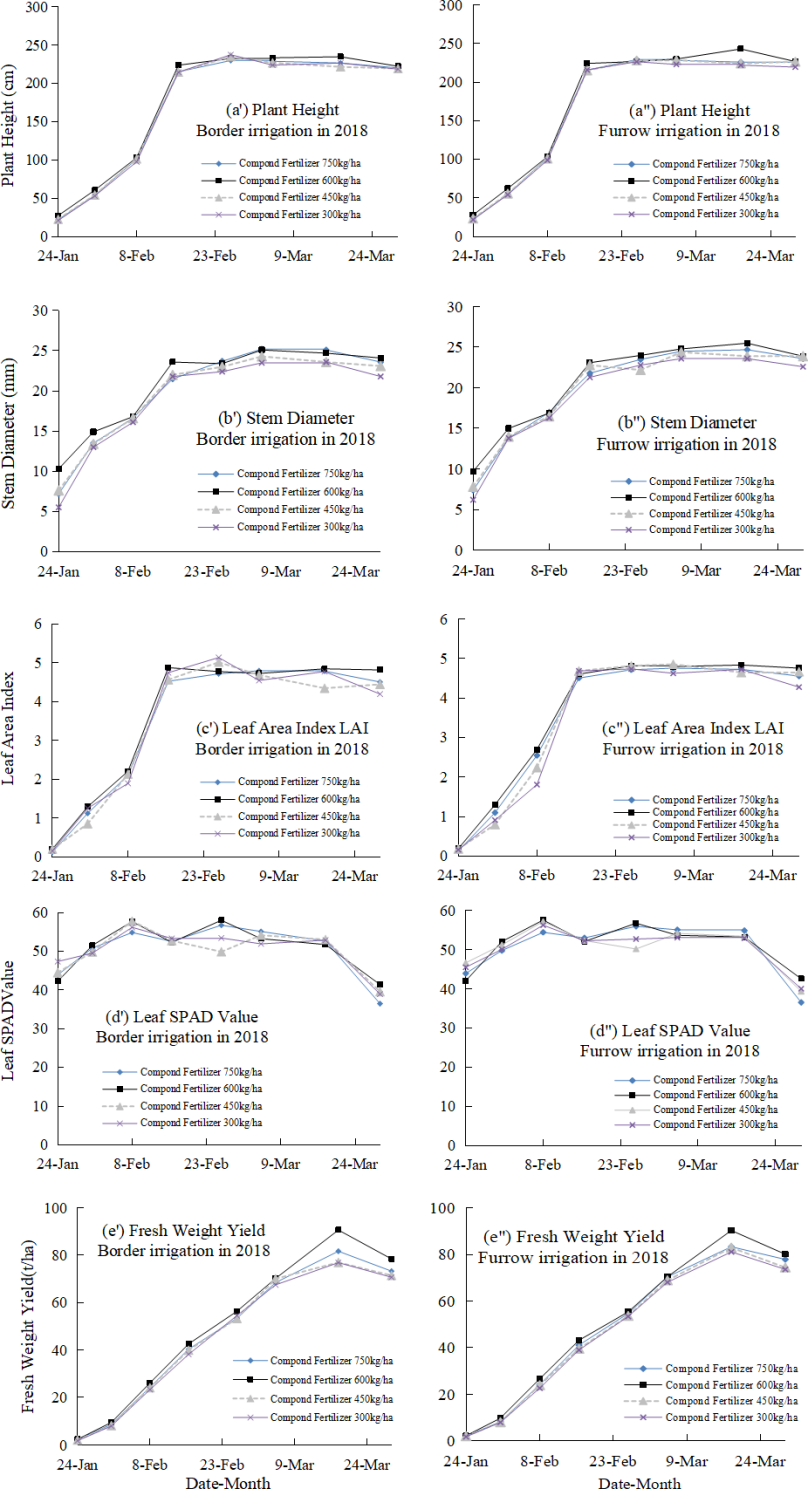
Plant height, stem diameter, leaf area index (LAI), SPAD value, and fresh weight yield of silage corn throughout the growing period in 2018, with data shown separately under border and furrow irrigation and different compound fertilizer application rates.

The changes of plant height, stem diameter, LAI, SPAD value, and fresh weight yield throughout the growing period under border and furrow irrigation and different compound fertilizer application rates in 2018 are shown in Fig. 3. The plant height, stem diameter at harvest, LAI at harvest under furrow irrigation was 2.12%, 1.3% and 1.6%, respectively, higher than that under border irrigation. The fresh weight yield at harvest was 73.51 and 76.55 t ha^−1^ for border and furrow irrigation, respectively, and the fresh weight yield for furrow irrigation was 4.1% greater than that for border irrigation. The plant height under the 600 kg ha^−1^ fertilizer application rate was 0.90%, 0.67%, and 2.40% greater than that under the 750, 450, and 350 kg ha^−1^ treatments, respectively. The 600 kg ha^−1^ treatment promoted the maximum stem diameter, the highest LAI and the maximum leaf SPAD value. The fresh weight yields at harvest were 75.63, 79.31, 72.98, and 72.19 t ha^−1^ under compound fertilizer application rates of 750, 600, 450, and 350 kg ha^−1^, respectively. The fresh weight yield under the 600 kg ha^−1^ treatment was 4.9%, 8.7%, and 9.9% greater than that under the 750, 450, and 350 kg ha^−1^ treatments, respectively. The ANOVA revealed that fertilizer application rate had a significant effect on fresh weight yield at a *p* = 0.01 level, while irrigation type and the interaction between irrigation type and fertilizer rate did not significantly affect the fresh weight yield.

Correlation and regression analyses were used to analyze the relationship between fresh weight yield (*y*) and plant height (*x*_1_), stem diameter (*x*_2_), LAI (*x*_3_), and the SPAD value (*x*_4_) of silage corn (Table 3). The correlations between fresh weight yield and various growth characters of silage corn showed that all four growth indexes are positively associated with the fresh weight yield of silage corn, and the fresh weight yield was significantly correlated with plant height, LAI, and leaf SPAD value at a *p* < 0.01 level in 2018. The regression equations based on the results of regression analysis are shown below for 2017 and 2018, respectively:

(8) \begin{eqnarray*}y& =4.37{x}_{1}+57.04{x}_{2}+101.56{x}_{3}+2.44{x}_{4}-2035.33\end{eqnarray*}

(9) \begin{eqnarray*}y& =4.82{x}_{1}+0.72{x}_{2}+51.54{x}_{3}+5.41{x}_{4}-571.81\end{eqnarray*}

**Table 3:** Correlation coefficients between fresh weight yield and plant height, stem diameter, leaf area index (LAI), and leaf SPAD value of silage corn in 2017 and 2018.

Year	Sample number	Plant height	Stem diameter	LAI	SPAD value
2017	16	0.125	0.373	0.048	0.140
2018	32	0.516^**^	0.230	0.480^**^	0.464^**^

**Notes.**

** Significant at *p* < 0.01.

* Significant at *p* < 0.05.

LAI, leaf area index.

Further stepwise regression analyses were conducted, showing that the fresh weight yield (*y*) was significantly correlated with plant height (*x*_1_) in 2017 and with plant height (*x*_1_) and leaf SPAD value (*x*_4_) in 2018 (Table 4). The ANOVA results corresponding to the above regression equations are summarized in Table 5, and the multivariate regression model was significant at a *p* = 0.01 level in 2018.

**Table 4:** The results of stepwise regression analysis between fresh weight yield (*y*) and plant height (*x*_1_), stem diameter (*x*_2_), leaf area index (LAI) (*x*_3_), and leaf SPAD value (*x*_4_) of silage corn in 2017 and 2018.

Year	Variable	Coefficient	Standard error	T	Sig.	Regression coefficient	*R*	DW
2017	*x*_1_	4.353	0.055	78.464	0	0.997	0.997	1.232
2018	*x*_1_	3.092	0.553	5.594	0	0.709	0.998	1.232
	*x*_4_	7.234	3.157	2.291	0.029	0.029		

**Table 5:** Analysis of variance results corresponding to the regression equations shown in Eqs. (8) and (9), relating fresh weight yield (*y*) to plant height (*x*_1_), stem diameter (*x*_2_), leaf area index (LAI) (*x*_3_), and leaf SPAD value (*x*_4_) of silage corn in 2017 and 2018, respectively.

Year	Terms	df	Sum of squares	Mean square	*F*	Sig.
2017	Regression	4	78,097.53	19,524.38	2.467	0.106
	Residual	11	870,66.70	7,915.15		
	Total	15	165,164.23			
2018	Regression	4	86,000.80	21,500.20	5.184^**^	0.003^**^
	Residual	27	111,972.56	4,147.13		
	Total	31	197,973.36			

**Notes.**

** Significant at *p* < 0.01.

### Effects on water consumption and crop coefficient

The ET_0_, ET_a_, and *K*_c_ values of silage corn in 2017 and 2018 are listed in Table 6. The average ET_0_was 354.83 mm for the whole growth period of silage corn in 2017 and 2018, and there were only minor differences between the two experiment years. Overall, ET_0_ of the two experiment years was almost the same, but ET_0_ during crop development period was 11.71 mm higher in 2018 than 2017. In particular, the greater number of precipitation days and greater total precipitation during the crop development period in 2017 may have been linked to the lower ET_0_. The average ET_a_ was 78.79, 123.97, 58.58, and 261.33 mm for the crop development, mid-season, late-season, and whole growth periods of silage corn, respectively, in the two years. ET_a_ was 18.93, 20.68, and 37.22 mm higher in 2017 than in 2018 for the crop development, late-season, and whole growth periods of silage corn, respectively. The average *K*_c_ was 0.56, 1.04, 0.62, and 0.74 for the crop development, mid-season, late-season, and whole growth periods of silage corn, respectively, in the two years. *K*_c_was 0.18, 0.18, and 0.11 greater in 2017 than that in 2018 for the crop development, late-season, and whole growth periods of silage corn, respectively.

**Table 6:** The ET_0_, ET_a_, and *K*_c_ for the crop development, mid-season, late-season, and whole growth periods of silage corn in 2017 and 2018.

Year	Item	Crop development	Mid-season	Late-season	Whole growth period
2017	Days after sowing	0–30	31–60	61–91	0–91
	ET_0_ (mm)	135.65	120.55	97.62	353.82
	ET_a_ (mm)	88.25	122.77	68.92	279.94
	*K*_c_	0.65	1.02	0.71	0.79
2018	Days after sowing	0–34	35–65	66–95	0–95
	ET_0_ (mm)	147.36	117.20	91.28	355.84
	ET_a_ (mm)	69.32	125.16	48.24	242.72
	*K*_c_	0.47	1.07	0.53	0.68

The ET_a_ and *K*_c_ of silage corn under different irrigation types and fertilizer application rates in 2018 and corresponding ANOVA results are summarized in Table 7. The average ET_a_ was 71.56, 128.10, 49.32, and 248.98 mm for the crop development, mid-season, late-season, and whole growth periods of silage corn, respectively, under border irrigation and 72.49, 126.70, 52.36, and 251.55 mm, respectively, under furrow irrigation. Additionally, the average *K*_c_ was 0.49, 1.09, 0.54, and 0.70 for the crop development, mid-season, late-season, and whole growth periods of silage corn, respectively, under border irrigation and 0.49, 1.08, 0.57, and 0.71, respectively, under furrow irrigation. There were minor differences among the ET_a_ and *K*_c_ values of silage corn under different fertilizer application rates. The ANOVA summarized in Table 7 indicated that irrigation type and fertilizer application rate had no significant effects on the ET_a_ or *K*_c_ values of silage corn.

**Table 7:** The ET_a_ and *K*_c_ values for the crop development, mid-season, late-season, and whole growth periods of silage corn in 2018 under different irrigation and fertilizer application rate treatments, with the corresponding ANOVA results.

Irrigation type	Fertilizer rate (kg/ha)	Development (0–34 days)		Mid-season (35–65 days)		Late-season (66–95 days)		Whole period (0–95 days)	
		ET_a_ (mm)	*K*_c_	ET_a_ (mm)	*K*_c_	ET_a_ (mm)	*K*_c_	ET_a_ (mm)	*K*_c_
Border irrigation	750	69.01	0.47	136.60	1.17	49.78	0.55	255.39	0.72
	600	69.32	0.47	125.16	1.07	48.24	0.53	242.72	0.68
	450	79.52	0.54	122.68	1.05	53.80	0.59	256.00	0.72
	300	68.39	0.46	127.94	1.09	45.46	0.50	241.79	0.68
Furrow irrigation	750	71.17	0.48	127.01	1.08	51.95	0.57	250.13	0.70
	600	79.52	0.54	131.03	1.12	51.33	0.56	261.88	0.74
	450	69.01	0.47	129.79	1.11	50.71	0.56	249.51	0.70
	300	70.24	0.48	118.97	1.02	55.45	0.61	244.66	0.69
ANOVA *p*-value	Irrigation type	0.843^NS^		0.780^NS^		0.340^NS^		0.693^NS^	
	Fertilizer rate	0.765^NS^		0.662^NS^		0.923^NS^		0.642^NS^	

**Notes.**

NS, Non-significant.

** Significant at *p* < 0.01.

* Significant at *p* < 0.05.

### Effects on water and fertilizer use efficiency

**Figure 4:**
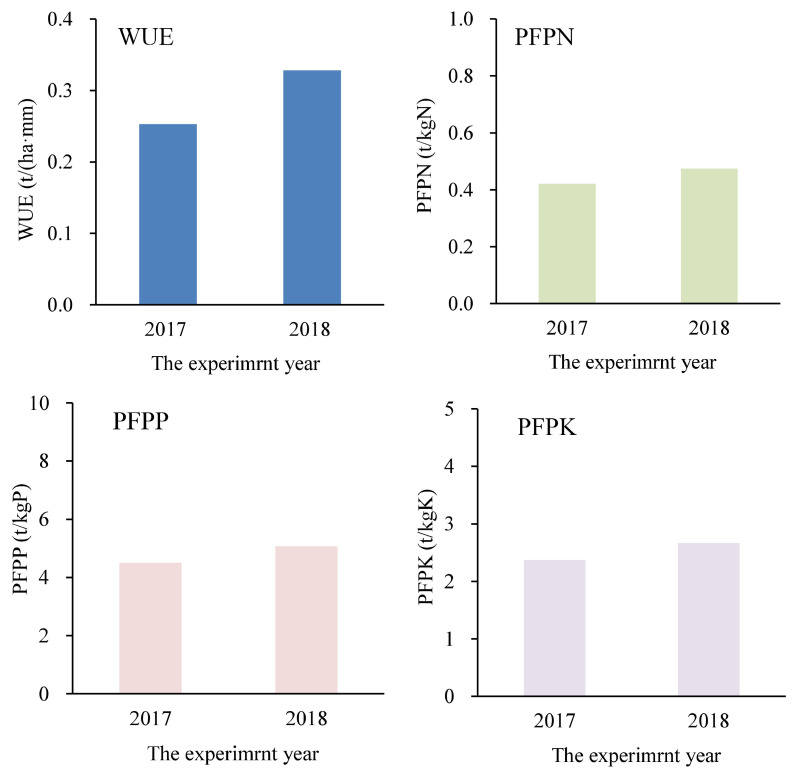
The water use efficiency (WUE) and partial fertilizer productivity of N (PFPN), P (PFPP), and K (PFPK) of silage corn in 2017 and 2018.

The WUE, PFPN, PFPP, and PFPK of silage corn in 2017 and 2018 are presented in Fig. 4 and Table 8. The range of WUE was 0.261–0.300, and its average was 0.277. The WUE in 2018 was 32% higher than that in 2017. The WUE under furrow irrigation was slightly higher than that under border irrigation, especially when the fertilizer application rate was 750, 450, or 300 kg ha^−1^. The WUE under a fertilizer application rate of 600 kg ha^−1^ promoted the maximum WUE value of 0.300.

**Table 8:** The water use efficiency (WUE) and partial fertilizer productivity of N (PFPN), P (PFPP), and K (PFPK) in 2018 under different irrigation types and fertilizer application rates and ANOVA results.

Treatment		WUE	PFPN	PFPP	PFPK
Irrigation type	Fertilizer application rate	t/(ha mm)	t/kg N	t/kg P	t/kg K
Border irrigation	750 kg/ha	0.288	0.351	3.750	1.973
	600 kg/ha	0.328	0.474	5.070	2.668
	450 kg/ha	0.291	0.592	6.327	3.329
	300 kg/ha	0.286	0.823	8.791	4.626
Furrow irrigation	750 kg/ha	0.314	0.374	4.000	2.104
	600 kg/ha	0.311	0.485	5.179	2.725
	450 kg/ha	0.298	0.591	6.315	3.323
	300 kg/ha	0.299	0.872	9.317	4.902
Analysis of variance *P*-value	Irrigation type	0.487^NS^	0.156^NS^	0.156^NS^	0.156^NS^
	Fertilizer application rate	0.317^NS^	0.000^**^	0.000^**^	0.000^**^

**Notes.**

NS, Non-significant.

** Significant at *p* < 0.01.

* Significant at *p* < 0.05.

The average PFPN, PFPP, and PFPK values were 0.570, 6.094, and 3.206, respectively. The PFPN, PFPP, and PFPK of silage corn in 2018 was a little more than that in 2017 because the fresh weight yield in 2018 was slightly higher. The PFPN, PFPP, and PFPK values under furrow irrigation were equal to or slightly higher than that under border irrigation. The PFPN, PFPP, and PFPK values were gradually increased when the fertilizer rate decreased from 750 to 300 kg ha^−1^ because the fresh weight yield among different fertilizer rates differed relatively little. ANOVA indicated that fertilizer application rate had a significant effect on PFPN, PFPP, and PFPK at the *p* < 0.01 level, and irrigation type had no significant effect on WUE.

## Discussion

Precipitation is the most important determinant of the irrigation schedule. Both 2017 and 2018 were normal hydrological years for the study site, but there was 62.9 and 19.68 mm more precipitation in 2017 than in 2018 during the crop development and mid-season growth periods, respectively (Fig. 1). The difference in precipitation patterns meant that no irrigation was utilized in 2017, while only 70 mm of irrigation was applied after sowing in 2018. As a consequence of different precipitation patterns and irrigation schedules, the silage corn appeared to grow slightly taller and thicker with a larger leaf area in 2017, though the fresh weight yield was 9.7% lower, relative to 2018 (Fig. 2). The results of regression analyses showed that the fresh weight yield of silage corn was significantly associated with plant height, LAI, and leaf SPAD value (Tables 3–5). Under the different precipitation patterns and irrigation schedules, the ET_a_ of silage corn was 37.22 mm higher in 2017 than 2018, and ET_a_ was 18.9 and 20.7 mm higher in 2017 than 2018 during the crop development and mid-season growth periods, respectively, although there was only a small difference in ET_0_ between 2017 and 2018 (Table 6). The larger fresh weight yield and the lower ET_a_ were consistent with the higher WUE in 2018, which was 32% larger than that in 2017 (Fig. 3). This suggested that relatively low irrigation during the crop development period of silage corn can promote high yield and higher WUE and fertilizer use efficiency, provided there is sufficient water to ensure the emergence of seedlings in the NPC and similar agricultural regions.

According to the 2018 results, furrow irrigation promoted growth compared with border irrigation under the same irrigation amount, but there was no significant difference in fresh weight yield, ET_a_, or WUE between irrigation treatments (Figs. 2 and 4, Table 4). Border irrigation is widely used in corn cultivation in the NCP, and thus, adoption of the potentially more effective furrow irrigation would require changes in the supporting machinery and technology in the region. Based on this issue, further research should be conducted to confirm the superiority of furrow irrigation in silage corn cultivation in the NCP. The previously documented advantages of furrow irrigation, such as water conservation and increased crop yields (Horst et al., 2005; Kefa et al., 2013), were not observed in this study.

The fertilizer application rate of 600 kg ha^−1^ was associated with slightly higher growth indexes and fresh weight yields compared with the rates of 750, 450, and 300 kg ha^−1^ (Fig. 2). The fertilizer application rate had a significant effect on fresh weight yield, PFPN, PFPP, and PFPK of silage corn, but did not affect ET_a_ or WUE of silage corn (Figs. 2 and 4, Table 4). In the NPC, the fertilizer use efficiency of crops is not particularly high (Zhang et al., 2008), so the determination of suitable fertilizer rates is vital to increasing crop use efficiency, decreasing soil fertilizer residue and limiting pollution of water bodies and groundwater. For the experimental field in this study, a fertilizer rate of 600 kg ha^−1^ was optimal for silage corn production.

## Conclusion

Supplementary irrigation is generally required in the NCP, and it is the primary method of making full use of precipitation resources. Even in normal hydrological years, variation in precipitation patterns can require the use of different irrigation schedules. Influenced by the combined effects of different precipitation patterns and irrigation schedules, the growth, yield, ET_a_, WUE, and fertilizer use efficiencies of silage corn were observed to differ between 2017 and 2018. Provided there is sufficient water to ensure the emergence of seedlings, relatively low irrigation at the crop development period of silage corn can promote high yields and higher WUE and fertilizer use efficiency in the NCP. Furrow irrigation promoted growth compared with border irrigation under the same irrigation amount, but had no significant effect on fresh weight yield, ET_a_, or WUE in this study. For the experiment field in this study, a compound fertilizer rate of 600 kg ha^−1^ was optimal for silage corn production. However, the advantages of furrow irrigation, such as water conservation and increased crop yields, was not observed in this study, and thus, further study on furrow irrigation in the NCP is merited.

##  Supplemental Information

10.7717/peerj.18315/supp-1 Supplemental Information 1 Data

## Additional Information and Declarations

### Data Availability

The following information was supplied regarding data availability: The raw measurements are provided in the Supplementary File.
